# Coliform pyosalpinx as a rare complication of appendicectomy: a case report and review of the literature on best practice

**DOI:** 10.1186/1752-1947-2-97

**Published:** 2008-04-02

**Authors:** Deepak Singh-Ranger, Abayomi Sanusi, Ishrak Hamo

**Affiliations:** 1 Department of General Surgery, Queen's Hospital, Rom Valley Way, Romford, Essex, UK

## Abstract

**Introduction:**

Coliform pyosalpinx is a rare entity. We report a case that occurred three months after appendicectomy for gangrenous appendicitis. There follows a literature review on best practice for the treatment of pyosalpinx.

**Case presentation:**

A seventeen year old girl presented with an acute abdomen three months after an appendicectomy for gangrenous appendicitis. Intraoperative findings were bilateral pyosalpinx treated by aspiration, saline and Betadine irrigation and intravenous antibiotics.

**Conclusion:**

Microbiological analysis of the pus revealed *Escherichia coli *and anaerobes. Chlamydia and Candida were not isolated. This is the first known reported case of Coliform Pyosalpinx following appendicectomy. The best treatment does not necessarily involve salpingectomy especially in women of reproductive age where fertility may become compromised.

## Introduction

Pyosalpinx, in the majority of cases, is a sequela of pelvic inflammatory disease. The ramifications of this condition are important and include tubal infertility and ectopic pregnancy [1]. There have been cases where a non-sexually transmitted cause for pyosalpinx has been described. Notable examples are pyosalpinx following *in vitro *fertilization [2] and infection by *streptococcus pneumoniae *[3] and coliforms [4]. Only one case of spontaneous coliform pyosalpinx has been published; that case involved a nine year old girl [5].

We report a case of coliform pyosalpinx in a seventeen year old girl following a recent appendicectomy. The best treatment for pyosalpinx in pre-menopausal females is discussed.

## Case presentation

A seventeen year old girl presented as an emergency with a two-day history of lower abdominal and back pain. She experienced rigors and appetite loss but no nausea, vomiting, dysuria, cystitis or vaginal discharge. Three months previously, she had undergone immediate appendicectomy for a gangrenous retrocaecal appendix. Other intraoperative findings at the time were a macroscopically normal right ovary and fallopian tube.

There was no history of recent sexual activity or pelvic inflammatory disease. Menstrual cycles were regular and every 28 days and the patient was mid-cycle at the time of presentation.

**Figure 1:**
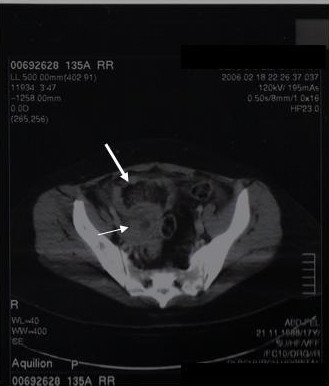
Emergency computerized tomographic scan: a right ovarian mass is visualized.

**Figure 2:**
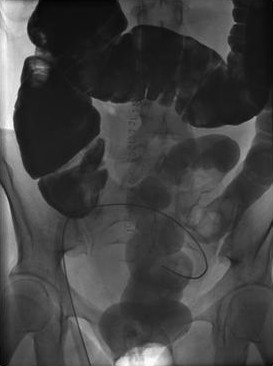
Postoperative Gastrografin enema did not show an enterotubal fistula.

On examination, she had a temperature of 38.5°C, pulse of 100 beats per minute and blood pressure of 114/59. Lower abdominal rebound tenderness, guarding and absent bowel sounds were present. The patient had a leucocytosis of 16.4 × 10^9^.l^-1 ^and C-reactive protein concentration of 322 mg.l^-1^. A pregnancy test was negative and an emergency computerized tomographic scan showed a complex pelvic mass associated with or near to the right ovary and overriding, but not connected to the uterus (Figure 1). She subsequently underwent an emergency laparotomy. The right fallopian tube was found in the midline above the uterus. It was grossly enlarged, measuring 10 × 5 cm, with multiple necrotic areas oozing pus. The fimbrial end was oedematous with a radius of 2 cm. The left fallopian tube was slightly enlarged and was found postrolateral to the uterus, adherent to the sigmoid colon by fibrinous adhesions. There was no visible enterosalpinx fistula and no appendicular stump leak. The left salpinx was released by blunt dissection and pus drained from both fallopian tubes by retrograde "milking". Both tubes were irrigated generously with a 0.9% saline and Betadine mixture. Microbiological analysis of the pus revealed *Escherichia coli *and anaerobes but not Chlamydia or Candida *spp*. A postoperative Gastrografin enema did not reveal an occult fistula (Figure 2).

The patient was treated postoperatively with intravenous Co-Amoxiclav and Metronidazole for a week and made an uneventful recovery. However, she now faces the long-term sequelae of potential infertility, ectopic pregnancy and chronic pelvic pain.

## Discussion

Coliform pyosalpinx is very rare, and coliform pyosalpinx following gangrenous appendicitis treated by appendicectomy has not been reported in the literature. This is the first report ever of this disease entity.

Pyosalpinx following appendicectomy may be one explanation for the small association between perforated appendicitis and sterility [6,7]. When encountered, it is vital for the trainee surgeon to be aware of the best treatment, with the least morbidity. This encompasses a wide range of interventions varying from intravenous antibiotics, laparoscopic aspiration or laparoscopic salpingotomy with saline irrigation, image-guided aspiration and/or drainage [8,9] to salpingectomy. The latter should be considered as last resort in premenopausal females. Repeat laparoscopy of patients who have undergone irrigation have shown no recurrence [10]. A randomized trial has shown that transvaginal sonographic drainage with intravenous antibiotics produces a faster resolution of symptoms than intravenous antibiotics alone; hospital stay and need for surgery were also lower in the study cohort.

The role of transvaginal drains and the effect of intra-fallopian antibiotic instillation on fertility still remains unclear.

One possible way to assess fertility is by performing a repeat diagnostic laparoscopy. This may demonstrate tubal features (e.g. occlusion, adhesions) that are linked to infertility [11,12]. The ideal time for the procedure is varied and ranges from between two to 33 weeks [13,14]. Tubal function may also be assessed by salpingography and/or salpingoscopy. A "cobblestone" appearance of the tubal mucosa is suggestive of patchy loss and damage to ciliated mucosal cells [13].

In premenopausal females, salpingectomy or laparotomy is not encouraged as subsequent infertility is said to be high [14].

In summary, coliform pysosalpinx may be a complication of acute gangrenous appendicitis and/or may follow appendicectomy. If diagnosed preoperatively sonographic or laparoscopic drainage is advocated. The small risk of infertility following open appendicectomy for perforated or gangrenous appendicitis may also be one argument for all premenopausal females to undergo a laparoscopic procedure for this condition.

## Conclusion

This is the first documented case of coliform pyosalpinx following appendicectomy for gangrenous appendicitis. It may be one reason for the association between perforated appendicitis and sterility [5,6]. In order to decrease the risk of infertility, minimally invasive treatment options should be used which endeavour to preserve the fallopian tubes in young females. Tubal patency and mucosal architecture can be assessed subsequently, by salpingography and salpingoscopy. Repeat diagnostic laparoscopy may also be useful in assessment of premenopausal females who have had appendicectomy but who are unable to conceive.

## Competing interests

The author(s) declare that they have no competing interests.

## Authors' contributions

DSR was involved in postoperative care during both admissions and drafted the manuscript. AZ obtained the Gastrografin radiological images, participated in revising the manuscript and was involved in the postoperative care during the second admission. IH was the consultant in-charge of the patient, performed the second operation, and has given approval of the manuscript.

## Consent

Written informed consent was obtained from the patient for publication of this Case report and accompanying images. A copy of the written consent is available for review by the Editor-in-Chief of this journal.
